# Towards a comprehensive tobacco-cessation approach: A pilot-training using simulation based-learning among medical students in Türkiye

**DOI:** 10.18332/tid/170278

**Published:** 2023-09-25

**Authors:** Dilek Karadoğan, Cüneyt Ardıç, Tahsin Gökhan Telatar, Yalçın Kanbay, Ekrem Kayaalp, Muhammet Ali Dedecan, Meltem Puşuroğlu, Songül Özyurt, Neslihan Özçelik, Bilge Yılmaz Kara, Nadir Emlek, Atilla Topçu, Sinan Saral, Kerem Uzun, Esin Bilgin Konyalıhatipoğlu, Elvin Karahacıoğlu Madran, İsmail Kavak, Hasan Göksun, Ünal Şahin, Sofia Belo Ravara

**Affiliations:** 1 Department of Chest Diseases, School of Medicine, Recep Tayyip Erdoğan University, Rize, Türkiye; 2 Department of Family Medicine, School of Medicine, Recep Tayyip Erdoğan University, Rize, Türkiye; 3 Department of Public Health, School of Medicine, Recep Tayyip Erdoğan University, Rize, Türkiye; 4 Department of Psychiatric Nursing, Faculty of Health Sciences, Artvin Coruh University, Artvin, Türkiye; 5 School of Medicine, Recep Tayyip Erdoğan University, Rize, Türkiye; 6 Department of Psychiatry, School of Medicine, Recep Tayyip Erdoğan University, Rize, Türkiye; 7 Department of Cardiology, School of Medicine, Recep Tayyip Erdoğan University, Rize, Türkiye; 8 Department of Pharmacology, School of Medicine, Recep Tayyip Erdoğan University, Rize, Türkiye; 9 Department of Physiology, School of Medicine, Recep Tayyip Erdoğan University, Rize, Türkiye; 10 Centro de Investigação em Ciências da Saúde, University of Beira Interior, Covilha, Portugal; 11 University Hospital Center of Cova da Beira, Covilha, Portugal; 12 Public Health Research Center (CISP), Nova University, Lisbon, Portugal

**Keywords:** medical students, tobacco cessation, medical school curriculum, simulation-based learning

**Dear Editor,**

Tobacco control (TC) is underpinned in two main strategies: preventing youth initiation and promoting tobacco cessation among users^1^. Healthcare professionals (HCPs), and specially physicians, should lead TC advocacy efforts, emphasize their role as TC leaders and exemplars, and disseminate TC and tobacco-cessation training, both at undergraduate and postgraduate level. However, most physicians are neither aware nor satisfactorily competent in TC roles^2^. Tobacco-cessation brief interventions can increase quit rates by 8%^3^, but their implementation in clinical practice remains poor^4^. In Türkiye, tobacco-cessation support is mainly provided by pulmonologists^5^; a survey reports that 41% of outpatients were asked about tobacco use, 31.8% were advised to quit, and 6% were referred to tobacco-cessation services^6^. Another cross-country European survey reports that less than 50% of smokers receive cessation advice or any support to quit; cessation support is scarce and inappropriate^7^. To scale-up a comprehensive tobacco-cessation approach, brief intervention should be embedded in health systems and systematically provided by all HCPs^4,8^. Medical School undergraduate training on tobacco cessation is crucial to shape attitudes and engage new generations of physicians^2^. However, both students and educators state that the medical curriculum is insufficient regarding tobacco-cessation^9,10^. The international researchers we inspired and their perspectives on tobacco cessation and cessation education were influential in the realization of this project^7^.

The aims of the current project were to: evaluate TC knowledge among final year students; implement a pilot training using simulation-based learning methods; and gather students’ opinions and suggestions about it. Inspiring international researchers and their perspectives on tobacco cessation contributed to this project.

Our pilot TC Training Project was held on 23–25 September 2022, at the Clinical Simulation Training Center of Recep Tayyip Erdoğan University, and supported by The Scientific and Technological Research Council of Türkiye. Participants were 18 final year medical students from 7 Turkish Universities (87.5% females, aged 23–25 years, 82.4% never smokers) and 17 educators. The project contents were: tobacco epidemic, tobacco-induced diseases, and tobacco dependence treatment. Examples of tobacco-induced diseases were practiced in ‘body interact’ applications with virtual patients, while brief tobacco-cessation interventions (5As model: ask, advice, assess, assist, arrange; and the 5Rs model: relevance, risks, rewards, roadblocks, repetition)^11^ were conveyed using theoretical and simulated patient and objective structured clinical examination (OSCE) rooms.

Tobacco-cessation outpatient clinic applications, tobacco addiction treatment monitoring system, pulmonary function test, and carbon monoxide measurements were performed with simulated patients using OSCE rooms (Figure 1). An online pre-post questionnaire evaluating baseline and post-training participants’ knowledge was administered. Pre-test and post-test scores were compared with the related-samples Wilcoxon signed rank test.

**Figure 1:**
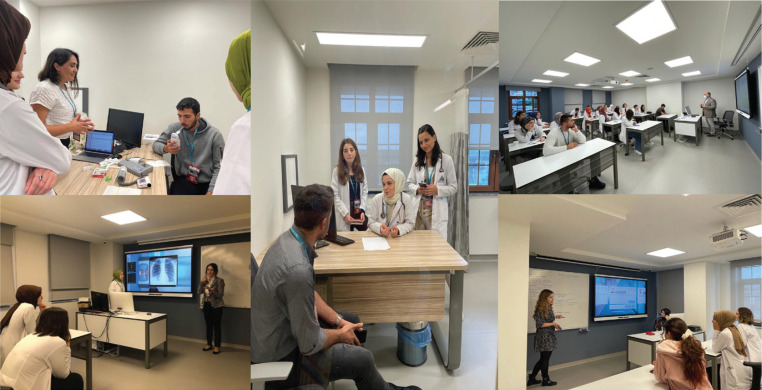
Education and training classrooms used throughout the course at the Recep Tayyip Erdoğan University Clinical Simulation Training Center during the project ‘Competent physician training program in tobacco control and cessation’, 23–25 September 2022, Rize, Türkiye Details from the web sites: http://rsim.erdogan.edu.tr/en and http://rsim.erdogan.edu.tr/tr/news-detail/tutun-kontrolunde-yetkin-hekim-egitimi-programi-23-25-eylul-2022-tarihlerinde-rsimde-gerceklestirildi/3703

Most participants reported attending only one annual TC lecture in their medical school; 41% considered TC contents insufficient in number and content. While the pre-test score ranged from 28 to 76 points (out of 110 total score), the post-test score ranged from 70 to 108 points; the pre-post test improvement rate was between 55% and 222%. The mean scores for the pre-test and post-test were 53.89 ± 16.47 and 94.54 ± 10.78, respectively (p<0.001). Students emphasized that the pilot learning experience on the 5As/5Rs model, both theoretical and practical, was very productive, in contrast to their medical education. In addition, they stated that the learning experience of tobacco-related diseases is valuable in establishing tobacco as the main preventable cause of lung and heart diseases, emphasizing the role of prevention. They also stated that applying smoking-cessation approaches in OSCE rooms in three different curriculum units (pulmonology, family medicine, psychiatry) was reinforcing both for practicing in different settings and for gaining practical skills.

This project is innovative in smoking cessation medical education, while using simulation-based learning methods in addition to examples of tobacco-related diseases given with virtual scenarios. In previous research combining a 4-hour theoretical and interactive practical course, students’ knowledge levels significantly increased compared to their base levels^12^. In our study, a significant increase was also observed in the pre-test and post-test levels. A previous study investigated how often tobacco control courses are given in medical education and the expectations of educators and students: it concludes that students demand to discuss the relationship between tobacco exposure and disease development in more detail. In addition, students stated that they would be more prompt in applying brief tobacco cessation practices such as 5As/5Rs to patients by explaining this association^9^. Another study emphasized that medical students were inadequately prepared to help patients change harmful behaviors such as tobacco use^10^. In our project, the link between tobacco use and the diseases was comprehensively discussed. Beside counselling skills, clinical benefits and the effects of quitting on the course of these diseases were discussed interactively.

In conclusion, integrating tobacco-cessation training in medical schools is feasible and well received by the students. In order to insert this practice in the medical education curriculum and to implement it consistently, educators’ as well as trainers’ motivation are also required. In this context, a main asset would be to receive support and contributions from international experts on tobacco control.

## Data Availability

Data sharing is not applicable to this article as no new data were created.
